# Oocyte-like cells induced from mouse spermatogonial stem cells

**DOI:** 10.1186/2045-3701-2-27

**Published:** 2012-08-06

**Authors:** Lu Wang, Jinping Cao, Ping Ji, Di Zhang, Lianghong Ma, Martin Dym, Zhuo Yu, Lixin Feng

**Affiliations:** 1 Institute of Medical Sciences, Shanghai JiaoTong University School of Medicine, 280 Chongqing S. Road, Shanghai, 200025, China; 2 Institute of Health Sciences, Shanghai Institutes for Biological Sciences, Chinese Academy of Sciences, Shanghai, China; 3 Department of Urology, General Hospital of Ningxia Medical University, Yinchuan, Ningxia, China; 4 Department of Biochemistry and Molecular & Cellular Biology, Georgetown University Medical Center, Washington, DC, 20057, USA

**Keywords:** Gametogenesis, Oocyte, PGC, Sex reversal, Spermatogonial stem cells

## Abstract

**Background:**

During normal development primordial germ cells (PGCs) derived from the epiblast are the precursors of spermatogonia and oogonia. In culture, PGCs can be induced to dedifferentiate to pluripotent embryonic germ (EG) cells in the presence of various growth factors. Several recent studies have now demonstrated that spermatogonial stem cells (SSCs) can also revert back to pluripotency as embryonic stem (ES)-like cells under certain culture conditions. However, the potential dedifferentiation of SSCs into PGCs or the potential generation of oocytes from SSCs has not been demonstrated before.

**Results:**

We report that mouse male SSCs can be converted into oocyte-like cells in culture. These SSCs-derived oocytes (SSC-Oocs) were similar in size to normal mouse mature oocytes. They expressed oocyte-specific markers and gave rise to embryos through parthenogenesis. Interestingly, the Y- and X-linked testis-specific genes in these SSC-Oocs were significantly down-regulated or turned off, while oocyte-specific X-linked genes were activated. The gene expression profile appeared to switch to that of the oocyte across the X chromosome. Furthermore, these oocyte-like cells lost paternal imprinting but acquired maternal imprinting.

**Conclusions:**

Our data demonstrate that SSCs might maintain the potential to be reprogrammed into oocytes with corresponding epigenetic reversals. This study provides not only further evidence for the remarkable plasticity of SSCs but also a potential system for dissecting molecular and epigenetic regulations in germ cell fate determination and imprinting establishment during gametogenesis.

## Background

Despite the different genotypes of germ cells in males with XY cells and females with XX cells, both types of germ cells share the same progenitors, namely, primordial germ cells (PGCs). The differentiation of PGCs into either the male or female phenotype takes place in the sex glands at later stages of embryonic development, and sexual differentiation of the germ cells is controlled by the somatic environment of the gonad rather than the sex chromosome constitution of the germ cells themselves [1-3]. Somatic mutation of sex-determining genes contributes to the sex reversal of XY germ cells to oogonia during gonad development; thus, the fate of XY male germ cells varies in response to environmental signaling in the gonad [4]. A few recent studies have demonstrated that spermatogonial stem cells (SSCs), which are the progeny of PGCs/gonocytes, can be reprogrammed into embryonic stem-like cells *in vitro* without transgene manipulation [5-9], indicating that SSCs retain remarkable plasticity. In addition, XY embryonic stem cells (ESCs) can differentiate into oocytes in culture [10]. Therefore, it is interesting to know whether SSCs can be reprogrammed into female germ cells. Here, we report that SSCs can be converted into oocyte-like cells in culture.

## Results

### Oocyte-like cells derived from SSCs in culture

**Figure 1:**
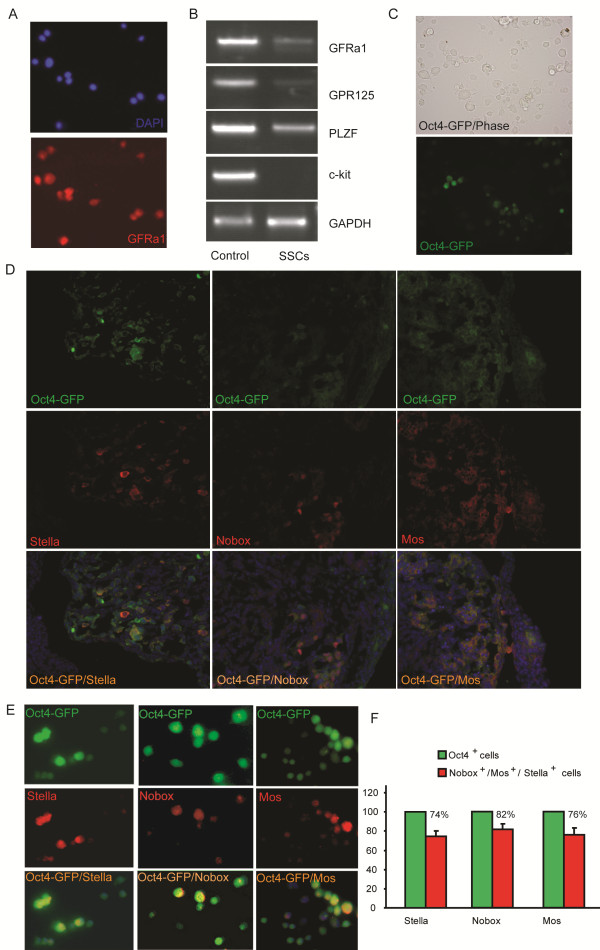
** Derivation of Oocyte-like cells from SSCs.(A)** Immunofluorescent staining with GFRa1 antibody of the magnetic-activated cell sorting (MACS)-isolated spermatogonial cells. **(B)** RT-PCR analyses of gene expression of GFRa1, PLZF, and c-kit in MACS-isolated spermatogonial cells (7 days old mouse testicular tissues were used for control of GFRa1 and PLZF, while 15 days old mouse testicular tissues were used for the c-kit positive control). **(C)** Phase contrast and fluorescence microscopy showing the expression of Oct4/GFP in cultured GFRa1^+^ SSCs from transgenic mice carrying the EGFP transgene driven by an Oct4 promoter (OG2 mice). **(D)** Immunofluorescence of ovarian sections of E17.5 embryos collected from pregnant OG2 females showing the co-expression of Stella (left), Nobox (middle), and Mos (right) with Oct4/GFP; Nuclei were stained by DAPI in blue. **(E)** Immunofluorescence showing that OG2-spermatogonia-derived Oct4/GFP cells differentiated into cells positive for Stella (left), Nobox (middle), and Mos (right) in culture of DMEM/F12 medium. **(F)** A statistical graph indicating the percentage of OG2-SSCs-derived Oct4/GFP cells that turned into oocytes positive for Nobox, Mos, or Stella.

**Figure 2:**
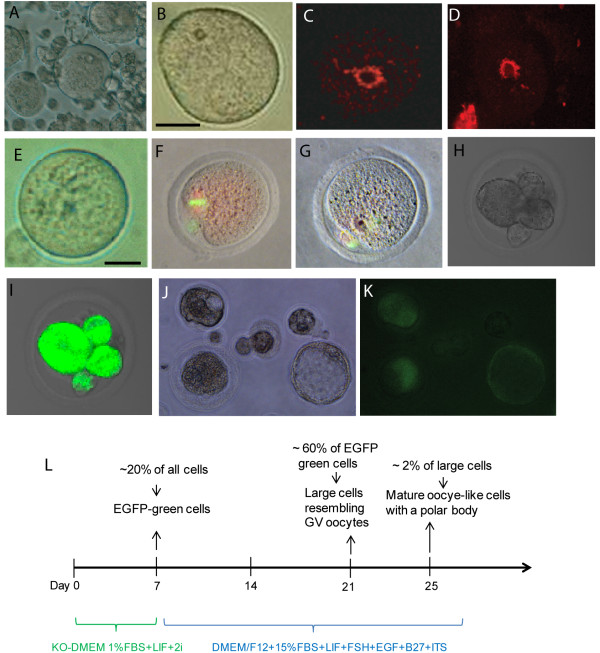
** Characterization of mature oocytes and embryos derived from spermatogonial stem cells (SSCs) in culture.(A and B)** Phase contrast microscopy of growing oocytes from the culture of SSCs (A), a SSCs-derived oocyte (SSC-Ooc) resembling that of germinal vesicle (GV) stage (B,scale bar = 30 μm). **(C and D)** Hoechst staining of a GV oocyte from a mouse ovary **(C)** and OG2-SSC-Ooc **(D**) showing a rim of chromatin around the nucleolus (the surrounded nucleolus). **(E)** Phase contrast microscopy of a fully-grown SSC-Ooc, scale bar = 30 μm. (F and G) YoYo1(green) and lamin B1 (red) staining of a control MII oocyte from a normal mouse ovary **(F)** and an oocyte with a polar body derived from SSC **(G)**. **(H and I)** A 4-cell embryo was generated from SSC-oocyte by ICSI with sperm from an OG2 mouse, from which GFP gene was carried by sperm and was expressed in the resulting embryo **(I)**. **(J and K)** Phase contrast microscopy of parthenogenetic embryos developed from OG2-SSC-oocytes in culture, Oct4/GFP from OG2 strain was expressed in the embryos **(K)**. **(L)** Schematic representation of the reprogramming conditions from SSCs to oocyte-like cells.

**Figure 3:**
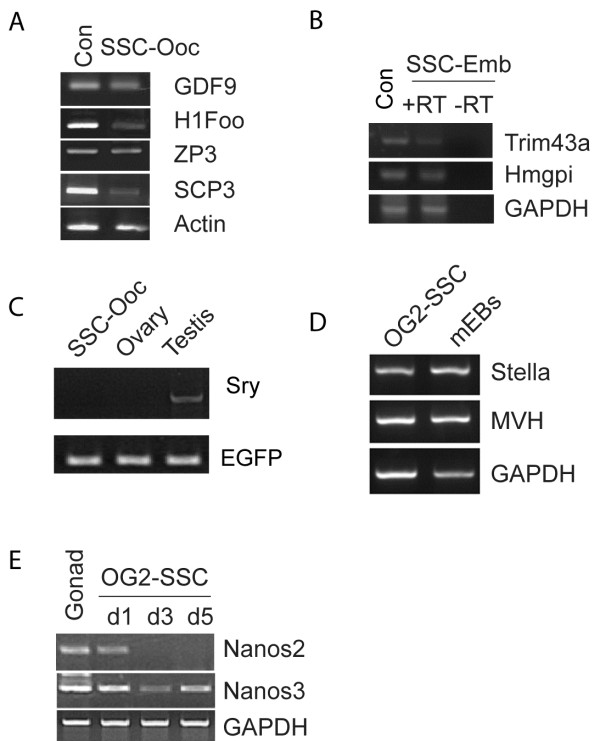
** Gene expression analysis of SSCs-derived oocyte (SSC-Oocs) and embryos derived from SSC-Oocs.(A)** RT-PCR analyses of the expression of GDF9, H1Foo, ZP3, and SCP3 in OG2-SSC-Oocs (Ovarian RNAs from a 30-day- old female were used as the control; ~150 SSC-Oocs of day 21 were collected for RT-PCR). **(B)** RT-PCR analyses showing the expression of Trim43a and Hmgpi in SSCs-derived embryos (embryos of E3.0 were collected from pregnant mice as a control; SSC-Emb, parthenogenetic embryos developed from SSC-Oocs). **(C)** PCR analysis of Sry gene in the genomic DNAs from mouse testis, ovary, and OG2-SSC-Oocs, EGFP was used as an internal control for all three genomic DNA samples prepared from OG2 mice. **(D)** RT-PCR analyses of the expression of Stella and MVH in the OG2-SSCs cultured for one week; mEBs (mouse embryoid bodies) were used as controls. **(E)** RT-PCR analyses of the expression of Nanos2 and Nanos3 in 5 days of culture of OG2-SSCs, mouse gonad tissue was used as a control.

We started with SSCs isolated by magnetic-activated cell sorting (MACS) with a GFRa1 [11] antibody and obtained GFRa1^(+)^ SSCs [12] (Figure 1A) from 8-day old OG2 transgenic mice (C57/B6 transgenic mice carrying the EGFP transgene driven by an Oct4 promoter). The isolated SSCs were further characterized by RT-PCR analyses for the positive and negative markers of SSCs (Figure 1B). We then cultured them in KO-DMEM medium containing 1% fetal bovine serum (FBS), 1,500 units/ml leukemia inhibitory factor (LIF) and 2i (2 μM SU5402 plus 3 μM CHIR99021) for one week, which synergize with the LIF signaling in pluripotency reprogramming [13,14]. Within the first week of culture, ~20% the Oct4/GFP expressing cells appeared (Figure 1C), indicating the dedifferentiation of SSCs under this culture condition. Our preliminary study demonstrated that DMEM/F12 medium supplemented with 15% FBS and LIF plus follicle-stimulating hormone (FSH), Epidermal growth factor (EGF), B27, and Insulin-Transferrin-Selenium-A (ITS) was useful in growing germ cell nuclear antigen( GCNA1)-positive germ cells from adult ovarian cells (Additional file 1: Figure S1A). Thus, we used this culture condition to test whether oogonial fate from the GFP-expressing cells can be induced. Under this culture condition for one more week, most of the GFP-expressing cells grew larger than SSCs. Interestingly, RT-PCR analyses indicated that oocyte-specific genes, including GDF-9 [15], Nobox [16], and Oogenesin [17], were expressed in the large cells (Additional file 1: Figure S1B). Thus, the oocyte-like cells appear to be derived from SSCs in culture. Furthermore, we used ovarian sections from E17.5 OG2 embryos as a control (Figure 1D) for the staining of Nobox, c-Mos, and Stella to examine the development of oocytes from GFP-positive cells (Figure 1E). We found that Oct4/GFP positive cells turned into Nobox-expressing cells (82%), Mos-expressing cells (76%), and Stella-expressing cells (74%) (Figure 1F). More interestingly, with extended culture by day 21, the SSC-derived oocytes (SSC-Oocs) grew larger (Figure 2A) and ~60% of these cells became oocytes resembling that of germinal vesicle (GV) stage (Figure 2B). They were further demonstrated by the formation of the ‘surrounded nucleolus’ (Figure 2C and D), a typical chromatin configuration in mouse oocyte at GV stage [18]. Among these growing oocytes, 10% grew to size similar to mature oocytes from mice (Figure 2E). Oocyte-specific markers, including H1Foo [19], zonapellucida 3 (ZP3), GDF-9, and SCP3 were expressed in these cells as demonstrated by RT-PCR analysis (Figure 3A). Meiotic and haploid SSC-Oocs were also identified by SCP3 staining and Giemsa stainingrespectively (Additional file 2: Figure 2). More surprisingly, ~2% SSC-Oocs with a structure like a polar body were also generated in culture (Figure 2F and G), and even gave rise to embryos (Figure 2J and K), most likely via parthenogenesis. The formation of embryos was further confirmed by the expression of genes from preimplantation embryos, such as *Hmgpi* and *Trim43a*[20,21] (Figure 3B). To test whether the SSC-Oocs were capable of being fertilized by sperm, we generated oocytes from BABL/c male pups using the same approach and used sperm from OG2 mice to carry out intracytoplasmic sperm injection (ICSI). In our early attempts, all SSC-Oocs died shortly after injection; The SSC-Oocs were very fragile, and they were severely damaged while being picked up and injected. To avoid this problem, we performed ICSI in the original culture dish without picking up SSC-Oocs before injection. We achieved success in fertilizing SSC-Oocs and obtained 4-cell embryos expressing GFP, which was carried by the sperm from OG2 mice (Figure 2H and I). Overall, with 53 attempts of ICSI, we obtained 5 embryos of early developmental stages after artificial activation with none beyond 4-cells. Collectively, the conditions for the oocyte induction from SSCs is summarized in Figure 2L.

### Primordial germ cells might be the intermediates from SSCs to Oocytes

To further confirm the genotype of these SSC-Oocs, we carried out a PCR analysis of *Sry*, which is located only on the Y chromosome, in the GFP-expressing larger SSC-Oocytes. We found that *Sry* was not present (Figure 3C), indicating that these SSC-oocytes should be of the XO karyotype and that YO cells died while growing due to the lack of whole X-linked genes.

**Figure 4:**
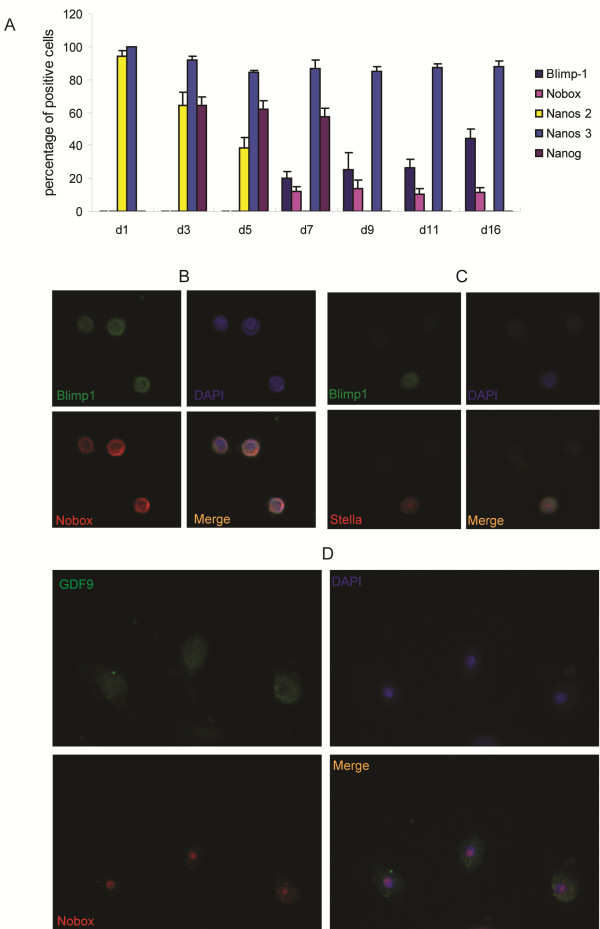
** Examination of the markers of PGCs and oocytes by immunofluorescent staining.(A)** Time course immunofluorescence analysis of the expression of Nanos2, Nanos3, Nanog, Blimp1, and Nobox, the percentage of positive cells for each corresponding marker at different days of culture was statistized. Data are means ± SE. (n = 4). **(B-D)** Co- immunofluorescence of Blimp1/Nobox **(B)**, Blimp1/Stella **(C)**, and GDF9/Nobox **(D)** in SSCs-derived oocyte.

Because ESCs can develop into oocytes [10] while cultured SSCs can be reverted to pluripotent cells [5-9], it has been proposed that cultured SSCs may be converted to pluripotent cells that subsequently develop into oocytes. A second hypothesis is that the cultured SSCs first revert to PGCs, which then develop into the oocytes. Moreover, the cultured SSCs can directly transdifferentiate into oocytes. To understand the potential mechanisms underlying the conversion of SSCs into oocyte-like cells, we examined the expression of genes related to PGC development in the early cultured SSCs. Interestingly, RT-PCR analyses showed that early cultured SSCs expressed PGC development-related genes, including *Stella* (*Dppa3*) [22], *Vasa* (*MVH*) [23], and *Nanos3*[24] (Figure 3D and E). Furthermore, we carried out a time-course staining cultured SSCs from BALB/c mice for PGC markers, including Nanos2, Nanos3, Nanog [25], and Blimp1 [26], and for the oocyte marker Nobox. Nanos2 [27] expression was lost within 3 days, while Nanos3 was maintained in more than 85% cells through two weeks of culture (Figure 4A and S3); the PGC markers, including Nanog and Blimp1, were induced around day 3 (Figure 4A and Additional file 3: Figure S3). Notably, Nanog expression was gradually lost before day 7, while Nobox expression started between day 5 and day 7 (Figure 4A and Additional file 1: Figure S3). We further confirmed the presence of oocytes by co-staining of multiple markers of oocytes. As shown in Figures 4B-D, Blimp1 positive cells expressed Nobox and Stella, while GDF9 positive cells expressed Nobox. Moreover, compact colonies of embryonic stem-like cells were never observed in our cultures. In addition, *E-cadherin*, which is highly expressed in mouse ESCs,was not detectable in these cells, and teratoma could not form from them (data not shown). These results indicate that ESC-like cells were less likely to form in this culture but PGCs and oocytes were produced. Therefore, the cultured SSCs probably developed into oocytes through PGC intermediates.

### Sex-specific imprint pattern and sex chromosome-linked gene activation are reversed during the conversion SSCs into oocytes

**Figure 5:**
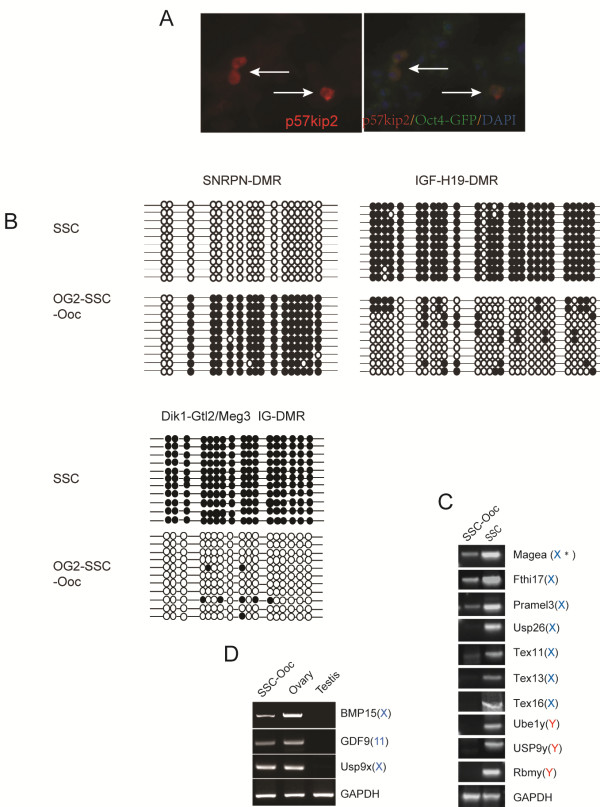
** Analysis of Sex-specific imprint pattern and sex chromosome-linked gene activation.(A)** Immunofluorescence of p57KIP2 in large cells derived from the OG2-SSC-Oct4/GFP cells (arrows). **(B)** Bisulfite genomic sequencing analyses of the DMR methylation status of parental origin-specific DNA methylation genes including Snrpn-DMR, Igf2-H19-DMR, and Dlk1-Meg3/Gtl2-DMR; filled ovals indicate methylated CpGs, and open ovals indicate unmethylatedCpGs (SSCs carry hypermethylation of Igf2-H19-DMR and Dlk1-Meg3/Gtl2-DMR but hypomethylation of Snrpn-DMR; this pattern was reversed in the OG2-SSC-Oocs). **(C)** Examination of the expression of sex chromosome-linked genes in OG2-SSC-Oocs: RT-PCR analyses showing that 7 testis-specific X-linked genes were significantly down-regulated (Magea, Fthi17, and Pramel3) or turned off (Usp26, Tex11, Tex13, and Tex16), three Y-linked genes (Usp9y, Ube1y, and Rbmy) were not expressed; GAPDH was used as the RT-PCR control. (D)RT-PCR analyses showing that oocyte-specific genes including X-linked genes (Usp9x and Bmp15) and autosomal gene GDF9 were turned on in OG2-SSC-Oocs; GAPDH was used as the RT-PCR control.

Parental imprints are established during gametogenesis and are essential for the function of gametes and the normal development of embryos. Thus, we were interesting to learn if imprinting reversals can be induced during the conversion of SSCs into oocytes. We examined the maternally-expressed imprinting gene *p57KIP2*[28] in the OG2-SSC-Oocs by immunofluorescence and found that *p57KIP2* was expressed in Oct4/GFP positive large cells (Figure 5A). Furthermore, we employed bisulfite genomic sequencing to examine the methylation status of the H19-, Snrpn-, and Dik-Gtl2/meg3-imprinting control regions in SSC-Oocs. We found that the DMR of maternally imprinted Snrpn was methylated, while the DMR of paternally imprinted Igf-H19 and Dik-Gtl2/meg3 were highly unmethylated, in comparison with freshly isolated SSCs from 8-day old mouse testes (Figure 5B). These results indicate that the epigenetic switching of imprints was associated with the transdifferentiation of SSCs into oocytes.

The activity of X chromosome-linked genes in male germ cells is different from that of female germ cells. The Y chromosome genes have been reported to be essential for spermatogenesis but not for oogenesis. Therefore, we addressed the gene activity status of sex chromosomes in the SSC-Oocs. We examined the expression of sex-dependent X- and Y-linked genes [29,30] and found that the X-linked testis specific genes were significantly down-regulated or turned off (Figure 5C and Additional file 4: Table S2), while oocyte specific genes including *GDF9*, X-linked *BMP15* and *Usp9x* were turned on (Figure 5D). The Y-linked genes were silenced (Figure 5C and Additional file 4: Table S2). The loss of the expression of Y-linked gene could be the result of the absence of the Y chromosome in the SSC-Oocytes since YO cells might die. These data indicate that the gene expression pattern of the X chromosome was changed in favor of the formation of oocytes from SSCs. Thus, following the process of SSCs dedifferentiation back to gonocytes/PGCs and transdifferentiation into oocytes, the X chromosome might be subjected to large scale changes in gene expression and epigenetic modifications. This hypothesis was supported by the reversal expression of *Usp9x* (Figure 5D and Additional file 2: Table S2), which is X-linked and expressed in both male and female embryonic germ cells, but turned off in male germ cells after birth [31]. Therefore, along with the morphological changes during the conversion of SSCs into oocytes, the epigenetic network was converted into the female germ cell form.

## Discussion

In mice, cellular pluripotency reprogramming mostly relies on the extrinsic signaling of LIF and the intrinsic factor Oct4; LIF signaling is sufficient in reprogramming of epiblast cells, in which Oct4 is not expressed, into pluripotent ESCs [32,33]. Oct4 in a defined culture condition can reprogram somatic cells into pluripotent cells [34]. It has also been revealed that a reversible path from stem cells to differentiation in the germ cell lineage exists [35-37]. In addition, a few studies have demonstrated that a small fraction of mouse SSCs can be reprogrammed back to embryonic stem-like cells [5-9]. Based on these findings, we thought as in the reprogramming epiblast cells into ES-like cells [32,33], the LIF signaling might trigger the dedifferentiation of SSCs. In the present study, we found that SSCs can dedifferentiate back into PGCs and transdifferentiate into oocyte-like cells when cultured in KO-DMEM medium containing 1% FBS, LIF plus 2i followed by DMEM/F12 medium supplemented with 15% FBS, LIF, FSH, EGF, B27, and ITS. This observation is consistent with earlier findings concerning the reprogramming capability of the LIF signaling in the presence of 2i [13,14] and further indicates the remarkable plasticity of SSCs in culture.

In our study, SSCs from 8 day-old testes were isolated and characterized as GFRa1^+^/PLZF^+^ but c-kit^-^. C-kit is a key marker of PGCs, and lack of c-kit expression excluded the presence of PGCs in the isolated cells. Thus, the isolated SSCs were unipotent. We used SSCs from 8 day-old OG2 male mice carrying EGFP transgene under an Oct4 promoter to trace the dedifferentiation. In situ, we can only observe green cells in testes of OG2 mice before postnatal day 6. We cultured SSCs from 8 days old testes, and found that ~20% off total SSCs were induced to express EGFP within one week, thereby indicating the dedifferentiation of SSCs. Despite of the presence of EGFP green cells that indicated they were positive of Oct4, we did not observe any ESC-like colonies formed from these cells. Furthermore, we found that *E-cadherin* expression was absent in them and teratoma could not form them in nude mice, indicating they were not ESCs but more like PGCs. This was further confirmed by the expression of Blimp1 and Nanog. These cells continued to grow with increasing size, expressed Stella, Nobox, and GDF9, and demonstrated morphology resembling that of oocytes. More interestingly, at around day 25 in culture, we observed ~2% of them developed into large cells with a polar body like MII oocytes. With our characterization of gene expression and morphology, we have clearly demonstrated that oocyte-like cells can be derived from SSCs via PGCs intermediates.

The imprinting patterns are established during gametogenesis with paternal imprints occurring during spermatogenesis and maternal imprints occurring during oogenesis. Defects in imprinting in gametes can give rise to severe problem in embryogenesis and predispose affected individuals to associated diseases after birth. To address if there were imprinting reversals in the conversion of SSCs into oocyte-like cells, we examined three key imprinting events including H19/Igf2, Snrpn, and Dik1-Gtl2/Meg3. It turned out that all three of them were switched to maternal status following the induction of oocyte-like cells from SSCs. Thus, in contrast to early report of germline-derived pluripotent stem (gPS) cells, which retain their original imprinting status [38], the reprogramming from SSCs to oocyte-like cells was accompanied by imprinting reversal. Therefore, SSCs possess both cellular and epigenetic plasticity and even give rise to oocyte-like cells *in vitro* in a manner similar to cases of sex reversal *in vivo*. Finally, we also demonstrated that a small number of SSC-Oocs were capable of being fertilized by sperm in vitro.

## Conclusions

Our study has demonstrated that SSCs possess the potential to be reprogrammed into oocyte-like cells in culture. If culture conditions are further optimized, for example, using 3D culture system with ideal supportive cell feeder [39], to develop oocytes of high quality from SSCs, such a germ cell fate switch system should provide a useful *in vitro* model to study epigenetic regulation in oogenesis and sex reversal, furthering our understanding of the mechanisms that establish imprinting during gametogenesis.

## Methods

### Ethics statement

This study was conducted in compliance with the National Institutes of Health *Guide for the Care and Use of Laboratory Animals* with the approval (SYXK-2003-0026) of the Scientific Investigation Board of Shanghai Jiao Tong University School of Medicine, Shanghai, China. Mice were euthanized by CO_2_ inhalation to ameliorate any suffering throughout these experimental studies.

### Cell culture

We used two enzymatic steps to isolate the adult ovarian germ cells from a 60-day old nursing BALB/c female mouse, whose male pups were used to isolate SSCs. The ovarian tissue was cut into small pieces followed by trypsin digestion for 5 minutes, then washed with DMEM/F12 (GIBCO, Grand Island, NY, USA) once followed by treatment with 0.1% bovine testicular hyaluronidase (Sigma Aldrich, St Louis, MO, USA) for 20 minutes. Cells were dispersed by pipette and suspended in DMEM/F12 medium containing 1% FBS (Biochrom AG, Berlin, Germany) and seeded onto 6 well plates. After 24 hours culture, the round cells, which were mostly on the top of plate-adhering cells, were collected by pipette and cultured in the DMEM/F12 medium containing 15% FBS, 1,500 units/ml LIF (ESGRO, Chemicon, Billerica, MA, USA), 0.5 IU/ml FSH, 10 ng/ml EGF (Invitrogen, Carlsbad, CA, USA), B27 (GIBCO), and ITS supplement (GIBCO).

SSCs were isolated from 8-day old OG2 mice using two-step enzymatic digestion followed by MACS (Miltenyi Biotech, BergischGladbach, Germany) using GFRa1 antibody, a goat anti-mouse antibody recognizing the C-terminus of the GFRa1 receptor (Santa Cruz Biotechnology, Santa Cruz, CA, USA), with a 1:200 dilution. Isolated SSCs were cultured in gelatin-coated 6-well plates with KO-DMEM medium containing 1% FBS,1,500 units/ml LIF, and SU5402 (2 μM, Calbiochem, La Jolla, CA, USA) plus CHIR99021(3 μM, Axon Medchem, Groningen, Netherland)-2i, for one week; After GFP positive cells appeared, DMEM/F-12 medium containing 15% FBS, 1,500 units/ml LIF, 100 microM 2-mercaptoethanol (GIBCO), FSH (0.5 IU/ml), EGF (10 ng/ml), B27, and ITS supplement was used for further culture.

### PCR, RT-PCR, and immunofluorescence

For PCR and RT-PCR, we used the primers listed in Additional file 5: Table S1. E 3.0 Embryos were collected from pregnant mice for analysis of the expression of Trim43a and Hmgpi. For immunofluorescence, the following antibodies were used: GFRa1 antibody (goat polyclonal ,Santa Cruz Biotechnology); PLZF antibody (rabbit polyclonal, Abcam, Cambridge, MA, USA); Anti-p57 Kip2 (Cell Signaling, Inc. Danvers, MA, USA); Nobox antibody (rabbit polyclonal, Abcam); Stella antibody (rabbit polyclonal, Abcam); Mos antibody (rabbit polyclonal, Santa Cruz Biotechnology); Nanog antibody (rabbit polyclonal, Abcam); Gamma-tubulin antibody (mouse monoclonal, Sigma-Aldrich); We utilized specific markers for nuclear envelope (lamin B1) and nucleic acids (YoYo1) to demonstrate the presence of polar bodies in oocytes, Lamin B1 antibody(1:100, Rabbit polyclonal, Abcam), and YOYO-1 (Molecular Probes, YOYO®-1 Iodide (491/509)), diluted in phosphate-buffered saline to make 2.4nM to use.

### Intracytoplasmic sperm injection (ICSI)

SSC-Oocs were rinsed thoroughly and kept in Hepes-CZB in original culture dish before injection. Adult OG2 mice were used as the donor for Oct4/GFP-carrying sperm. To retrieve sperm, seminiferous tubules were collected and put in Hepes-CZB. They were then cut into small pieces with a pair of fine scissors. A drop of the medium with tubule fragments was mixed with the same volume of Hepes-CZB containing 12% (w: v) polyvinylpyrrolidone and pipetted vigorously to release spermatozoa. Sperm were collected and injected into SSC-Oocs. Injected oocytes were activated by 30-min treatment with Ca^2+^-free CZB containing 5 mM SrCl_2_. Embryos were cultured in KSOM at 37°C in 5% CO_2_.

### Bisulfite methylation analysis

Genomic DNA was isolated from SSCs and SSC-Oocs. Bisulfite conversion was performed on a thermocycler using the QiagenEpiTect Kit (Qiagen, Hilden, Germany) according to manufacturer’s instructions, with two additional cycles (5 min at 99°C and 3 h at 60°C) at the end. Converted DNA was eluted in 40 μl of elution buffer, and a 5-μl DNA sample was then amplified with the following primer sets: *Snrpn*, AATTTGTGTGATGTTTGTAATTATTTGG and ATAAAATACACTTTCACTACTAAAA TCCACAA; *Igf2-H19*-DMR, GGAATATTTGTGTTTTTGGAGGG and TTAAACCCCAACCTCTACTTTT ATAAC; *Dlk1-Meg3/Gtl2*-DMR, GGTTTGGTATATATGGATGT ATTGTAATATAGG and ATAAAACACCAAATCTATACCAAAATATACC. PCR was performed in 25-μl reactions using 2.5 units of ExTaq under the following conditions (38 cycles): 1) 96°C for 15 seconds, 60°C for 30 seconds, and 72°C for 30 seconds for *Snrpn*; 2) 96°C for 15 seconds (hot start), 55°C for 30 seconds, and 72°C for 1 minute for *Igf2-H19*-DMR and *Dlk1-Meg3/Gtl2*-DMR. The amplified fragments were cloned into the pMD19-T vector (TaKaRa Biotech Co., Ltd) and then sequenced.

### Statistics

All experiments were performed 4 times, and data were expressed as means ± SE and analyzed by one-way ANOVA analysis. A value of *P < 0.05* was considered significant.

## Competing interests

The authors declare that they have no competing interest.

## Author’s contributions

PJ helped the cell culture, JC did Bisulfite methylation analysis, DZ and LM prepared animals and isolated germ cells, LW and ZY carried out most experiments, MD and LF designed the overall study, ZY and LF prepared the manuscript. All authors read and approved the final manuscript.

## Supplementary Material

Additional file 1 **Figures S1.** (A) Culture of ovarian germ cells from an adult BABL/c female in DMEM/F12 + 15% FBS + LIF showing that GCNA positive cells (red, identified by immunofluorescent staining with GCNA antibody provided from George Enders, University of Kansas Medical Center). (B) RT-PCR analyses showing the expression of GDF-9, Nobox, and Oogenesin in oocytes and SSC-derived Oocytes. Click here for file

Additional file 2 **Figure S2.** (A) Immunofluorescence of SCP3 showing a meiotic SSC-derived oocyte. (B) Giemsa staining demonstrates a SSC-derived haploid oocyte. Click here for file

Additional file 3 **Figures S3.** Time-course staining of the markers of primordial germ cells (PGCs) and oocytes. BALB/c SSCs were isolated and cultured in KO-DMEM for one week, then cultured in DMEM/F12 medium and subject to immunofluorescence staining at different times with antibodies against primordial germ cells (PGCs) and oocyte markers, including Nanos2, Nanos3, Nanog, Blimp1, and Nobox. Click here for file

Additional file 4 **Table S2.** In SSC-Oocs, X- and Y-linked testis specific genes were turned off, X-linked ovary specific genes were turned on. GDF9, an oocyte specific gene, was turned on too. Click here for file

Additional file 5 **Table S1.** PCR Primers. Click here for file
