# Supplementary figures and images for: Oocyte-like cells induced from mouse spermatogonial stem cells
Source: Cell Biosci. 2012 Aug 6;2:27. doi: 10.1186/2045-3701-2-27 (PMC3505744; doi:10.1186/2045-3701-2-27)

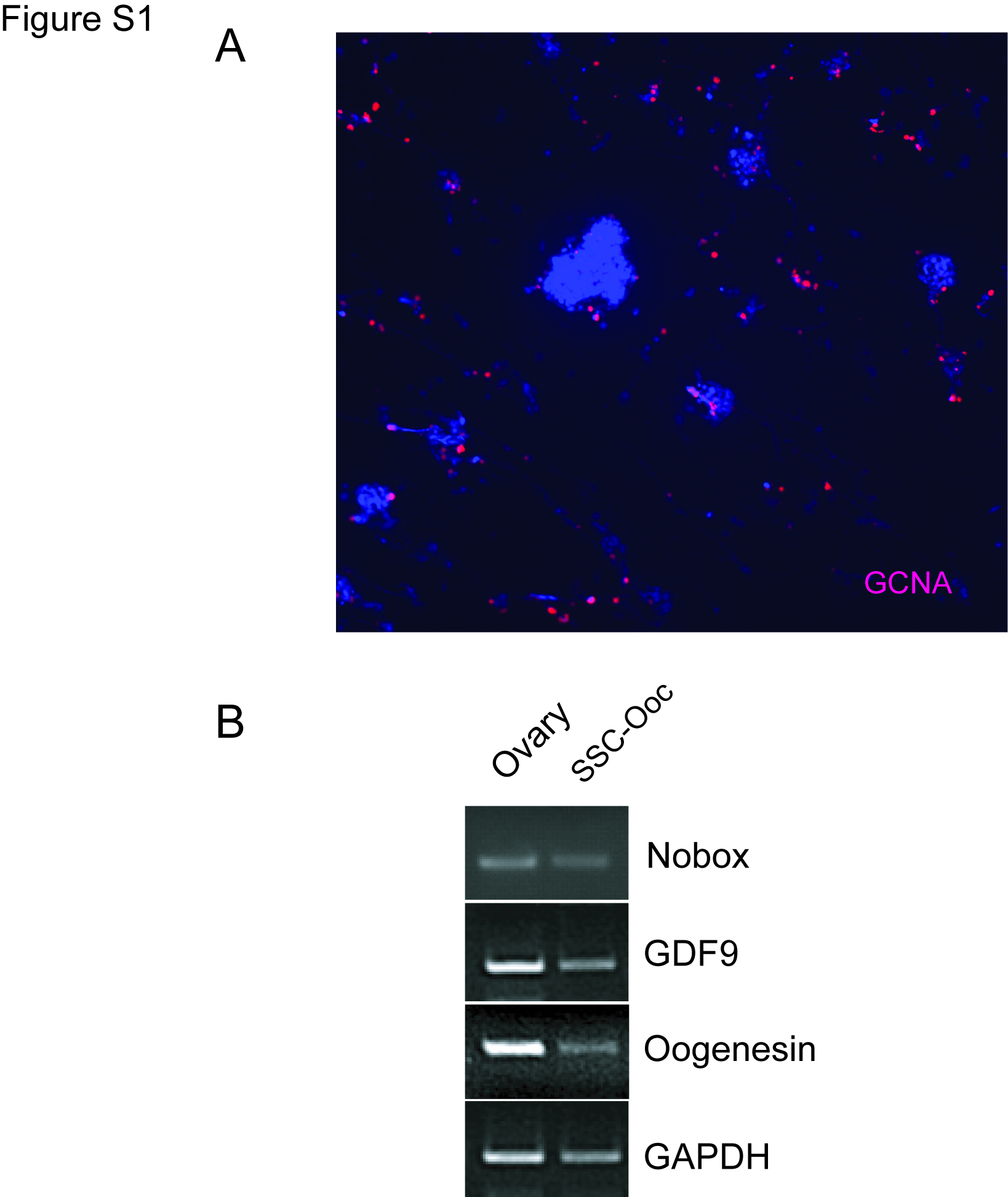

Supplement: Additional file 1 — Figures S1. (A) Culture of ovarian germ cells from an adult BABL/c female in DMEM/F12 + 15% FBS + LIF showing that GCNA positive cells (red, identified by immunofluorescent staining with GCNA antibody provided from George Enders, University of Kansas Medical Center). (B) RT-PCR analyses showing the expression of GDF-9, Nobox, and Oogenesin in oocytes and SSC-derived Oocytes. [file 2045-3701-2-27-S1.tiff]

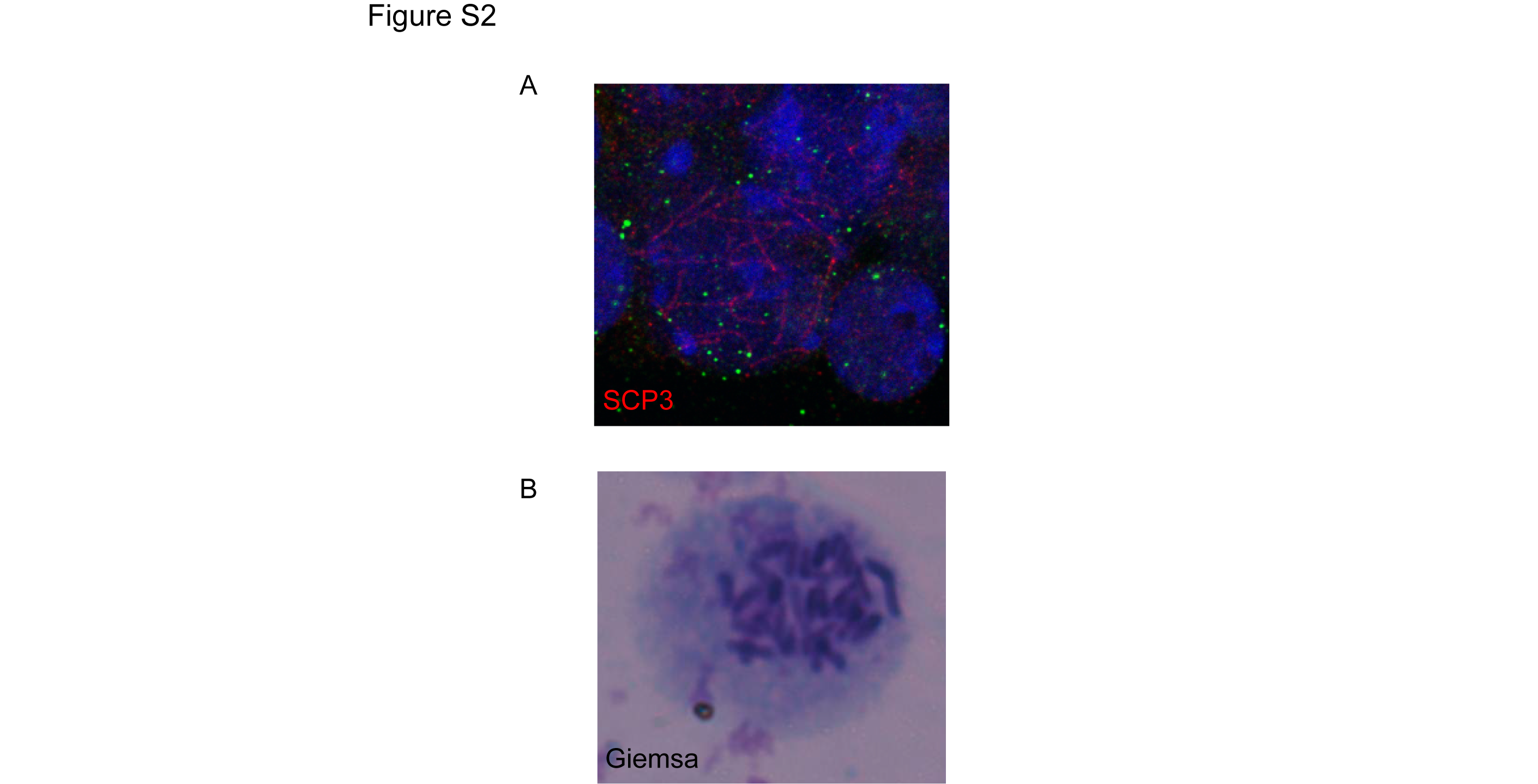

Supplement: Additional file 2 — Figure S2. (A) Immunofluorescence of SCP3 showing a meiotic SSC-derived oocyte. (B) Giemsa staining demonstrates a SSC-derived haploid oocyte. [file 2045-3701-2-27-S2.tiff]

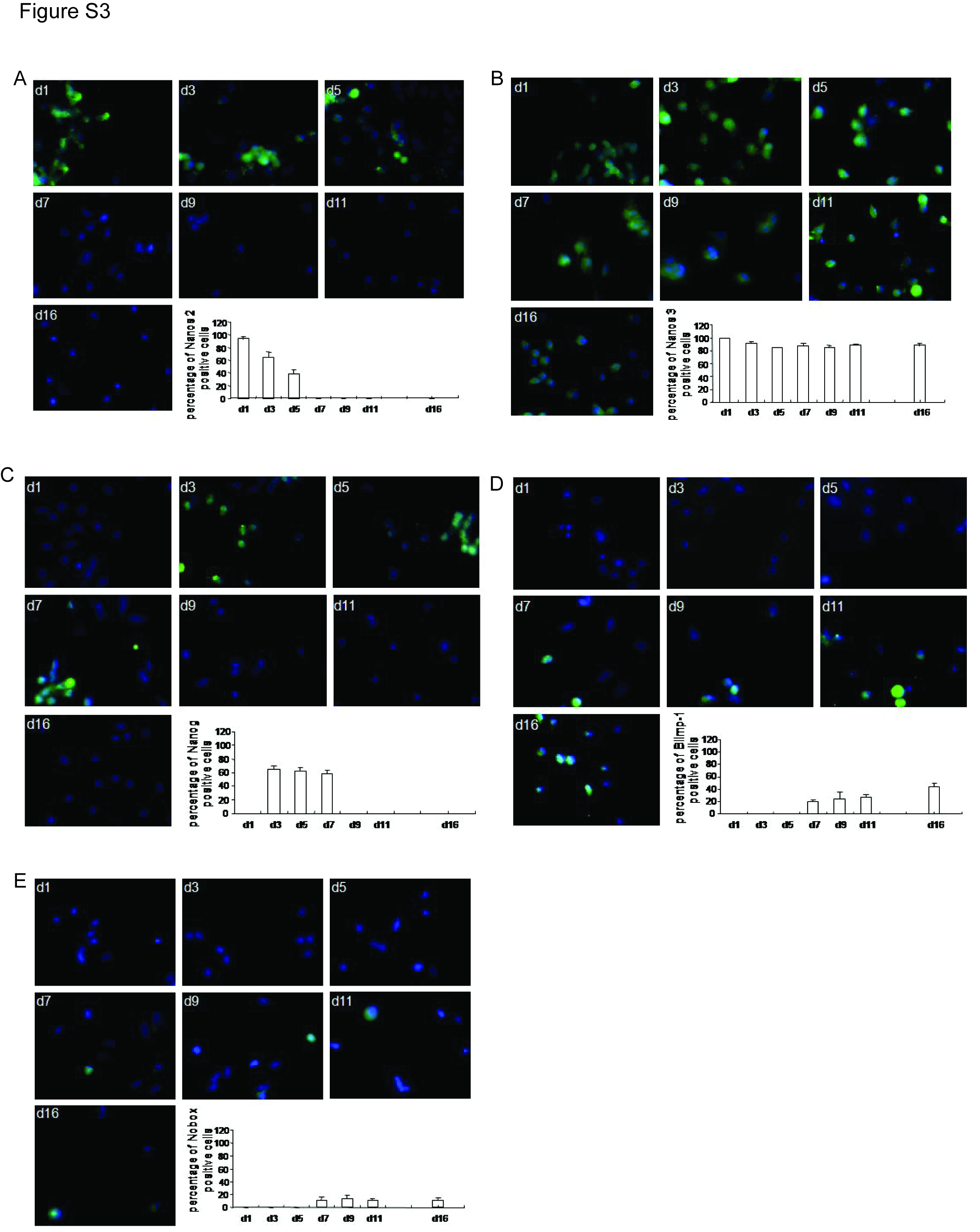

Supplement: Additional file 3 — Figures S3. Time-course staining of the markers of primordial germ cells (PGCs) and oocytes. BALB/c SSCs were isolated and cultured in KO-DMEM for one week, then cultured in DMEM/F12 medium and subject to immunofluorescence staining at different times with antibodies against primordial germ cells (PGCs) and oocyte markers, including Nanos2, Nanos3, Nanog, Blimp1, and Nobox. [file 2045-3701-2-27-S3.tiff]
