# Supplementary material for: Oocyte-like cells induced from mouse spermatogonial stem cells
Source: Cell Biosci. 2012 Aug 6;2:27. doi: 10.1186/2045-3701-2-27 (PMC3505744; doi:10.1186/2045-3701-2-27)
Supplement: Additional file 4 — Table S2. In SSC-Oocs, X- and Y-linked testis specific genes were turned off, X-linked ovary specific genes were turned on. GDF9, an oocyte specific gene, was turned on too. [file 2045-3701-2-27-S4.doc]

Table S2. In SSC-Oocs, X- and Y-linked testis specific genes were turned off, X-linked ovary specific genes were turned on. GDF9, an oocyte specific gene, was turned on too.

| Name of gene | Chromosome location | Testis | ovary | SSC-Ooc |
| --- | --- | --- | --- | --- |
| Rbmy | Y | +++++* |  |  |
| Usp9y | Y | +++++ |  |  |
| Ube1y | Y | +++++ |  |  |
| Magea | X | +++++ |  | + |
| Fthi7 | X | +++++ |  | + |
| Pramel3 | X | +++++ |  | + |
| Usp26 | X | +++++ |  |  |
| Tex11 | X | +++++ |  | +(?) |
| Tex13 | X | +++++ |  |  |
| Tex16 | X | +++++ |  |  |
| Usp9x | X |  | +++++ | +++++ |
| Bmp15 | X |  | +++++ | +++ |
| GDF9 | 11 |  | +++++ | ++++ |

*. + represents the RT-PCR indicated mRNA abundance of a given gene.
