# Supplementary material for: Oocyte-like cells induced from mouse spermatogonial stem cells
Source: Cell Biosci. 2012 Aug 6;2:27. doi: 10.1186/2045-3701-2-27 (PMC3505744; doi:10.1186/2045-3701-2-27)
Supplement: Additional file 5 — Table S1. PCR Primers. [file 2045-3701-2-27-S5.docx]

Table S1. PCR Primers

| Gene | Sequences |
| --- | --- |
| GFR1 | 5’-AGAAGCAGTTTCACCCAG-3’, 5’-ATCATCACCACCACCAT-3’ |
| PLZF | 5'-TCTGTCTGCTGTGTGGGAAG-3',5'-GTGGCAGAGTTTGCACTCAA-3' |
| c-kit | 5’-CCCGACGCAACTTCCTTA-3’,  5’-CGCTTCTGCCTGCTCTTC-3’ |
| GDF9 | 5’-ATCGAGTGCAGTGTCCGTAGGT-3’ 5’-TTCACTTGGTTTATGGCAACG-3’ |
| H1Foo | 5’-CCACCCGTTTCAAGTACCTGTTG-3’  5’-CCTCCTCTTCTGCCCTTTCTCC-3 |
| ZP3 | 5’-TTGAGCAGAAGCAGTCCAGC-3’ 5’-CGGTTGCCTTGTGGATGGTC-3’ |
| SCP3 | 5’-ATGATGGAAACTCAGCAGCAAGAGA-3’ 5’-TTGACACAATCGTGGAGAGAACAAC-3’ |
| Sry | 5’’-TTACAGCCTGCAGTTGCC-3’ 5’-GGTCATAGAACTGCTGTTGC-3' |
| Sox2 | 5’-ATGTATAACATGATGGAGACGGAGC-3’  5’-TCACATGTGCGACAGGGGCAGTGT**-**3**’** |
| Nanog | 5’-AAGTACCTCAGCCTCCAGCA-3’ 5’-GTGCTGAGCCCTTCTGAATC-3’ |
| Stella | 5’-GACGCTTTGGATGATACAGAC-3’ 5’-GGTCTTTCAGCACCGACAACA-3’ |
| MVH | 5’-ATGATGCGGGATGGAATACT-3’  5’-CACCACCTCCATCCTTCAGTATGA-3’ |
| Trim43a | 5’ATTCCATTCCTGGGCTGACT3’  5’CTGACTGAGGTGTAGCCGAAC3’ |
| Hmgpi | 5'-GTTGGGAGTTGGACTATGGAC-3'  5'-TGAACTGATTGGACACACACA-3' |
| actin | 5’-ACCAACTGGGACGATATGGAGAAGA-3’ 5’-CTCTTTGATGTCACGCACGATTTC-3’ |
| GAPDH | 5’-AAGGGCTCATGACCACAGTC-3’, 5’-ACACATTGGGGGTAGGAACA-3’ |
| Megea | 5’-CCACCACCTCAAATAAAGTGT-3’ 5’-CTCACTAAAGATCAGAGGGTA-3 |
| Fthi17 | 5’-ACTTTGACCGTGATGACGTG-3’  5’- AGTTTTGCTCCAGGAAATGGC-3’ |
| Pramel3 | 5’-GATCTGTGAGGCAGAATGAAG-3’  5’- CATTAAGCCATCTGGATCTGA-3’ |
| Usp26 | 5’-AATGTAACGAAGGGAGAAGTG-3’  5’-AGGCTTTGCCTTCTTATCGAG-3 |
| Tex11 | 5’-TATCAGATTCCCTGGAACTGG-3’  5’-ACCCTCAAAACAAGCTATG-3’ |
| Tex13 | 5’-ACCAGAGTTGGGAACAACTAA-3’  5’-CTGTTGTAGAGGGTAGAGGTT-3’ |
| EGFP | 5’CATGGTGAGCCGTCTTTCCA 3’  5’TTCAGGGTCAGCTTGCCGTA 3’ |
| Tex16 | 5’-AACTTGAAATATAGCACTGATGAGACA-3’  5’- GGGAATGTTTAGTGTCTAGGA-3’ |
| Ube1y | 5’-CTCTGAGTACATCCGTGG-3’ 5’-GCAATCCTGCTGAACTGC-3’ |
| USP9y | 5’-GGCAGGTTGCACATTCAC-3’5’-GTCTTCATTACCCTGCAAGATC-3’ |
| Rbmy | 5’-AACCGAAGTAACATATACTCA-3’  5’- ATCTGCTTTCTCCACGACCTC-3’ |
| Bmp15 | 5’-GTGGGGAGTGGTGCTTTTTA-3’ 5’-CTCACCATTTTCGCTCCAAT-3’ |
| Usp9x | 5’-GAGAACTGTGGCAAGGAAGC-3’  5’-CTAGGCTCCAGCCAGCATAC-3’ |
| Oogenesin | 5’- ACCTCAGGTGCCTGA AAA AG-3,  5’- CAGCAAGGC ACTGAACTGAA-3’ |
| Nanos2 | 5’- CCAGACTGTGTGTCTCAGACATTCC-3’  5’- CAGTGACGATCAGGATCAGTCTCTG-3’ |
| Nanos3 | 5’- GGCAAAGACACAGGA TGCTG-3’  5’- AGTCTCCAGCATGCCTGTGT-3’ |
| Nobox | 5’- GGACAAGGCCTATGTGTCCT-3’  5’- GGGTCCTGTACCCATGTTTT-3’ |
